# Ischemic stroke secondary to self-inflicted carotid sinus massage: a case report

**DOI:** 10.1186/s13256-021-02680-1

**Published:** 2021-02-23

**Authors:** Edgar R. Lopez-Navarro, Götz Greif, Carl-Albrecht Haensch, Adrian Ringelstein, Robert Larbig

**Affiliations:** 1 Department of Neurology, Kliniken Maria Hilf, Viersener Str. 450, 41063 Moenchengladbach, Germany; 2 Department of Neurology, Kliniken Maria Hilf, University of Witten/Herdecke, Moenchengladbach, Germany; 3 Department of Radiology and Neuroradiology, Kliniken Maria Hilf, Moenchengladbach, Germany; 4 Department of Cardiology, Kliniken Maria Hilf, Moenchengladbach, Germany

**Keywords:** Watershed-type stroke, Carotid stenosis, Neck massage, Carotid sinus, Carotid web

## Abstract

**Background:**

The risk of stroke after carotid sinus massage is greater if there is preexisting carotid stenosis or carotid plaques. We present the case of a patient with underlying 40% carotid stenosis, who developed a watershed stroke after a self-neck massage in our stroke unit. We show a well-documented case with magnetic resonance images before and after the neck massage. We report a case of a watershed brain infarct after a self-massage of the carotid sinus, with preexisting carotid artery stenosis. Neck massage continues to be a significant cause of stroke and should therefore not be performed by patients. Clinicians must be aware of the implications of a carotid sinus massage in both the outpatient and inpatient settings.

**Case presentation:**

We admitted a 58-year-old white male patient, with no relevant medical history, to our department with a brain stem infarct. During his stay at our stroke unit, the patient performed a self-neck massage with consecutive bradycardia and asystole, resulting in left-side hemiparesis. The underlying cause of the hemodynamic stroke is believed to be secondary to this intensive neck massage performed by the patient. The patient also suffered from unknown right internal carotid artery stenosis.

**Conclusion:**

Clinicians and patients must be aware that neck massage can lead to ischemic stroke. We postulate that repetitive impaired cardiac output can lead to a hemodynamic (watershed-type) stroke.

## Background

To the best of our knowledge, the only reports of ischemic stroke after carotid sinus (CS) massage published thus far are cases occurring after therapeutic or diagnostic CS massage, and one case of a patient with multiple cerebral emboli due to a free-floating thrombus secondary to a neck massage [1].

## Case presentation

A 58-year-old, right-handed white male presented to our emergency room after he woke up with numbness on the right side of the face and in the right arm, dysphonia, dysarthria, and dysphagia. His past medical history was only significant in terms of arterial hypertension. The patient was an employee in a manufacturing plant; personal habits included occasional alcohol consumption, with no history of tobacco or drugs. At the time of admission he was on aspirin 100 mg daily (indication was unclear). The family history was unremarkable. In the emergency room, the initial systolic blood pressure was 223 mmHg, heart rate was 85 beats per minute, and the body temperature was 36.6 °C. In the neurological examination we found a right sensory brachiofacial syndrome, moderate to severe dysarthria and dysphonia, and dysphagia, with National Institutes of Health Stroke Scale score of 3. Based on suspected brainstem infarct, and following our institutional wake-up stroke protocol, magnetic resonance imaging (MRI) was performed, which showed an acute ischemic lesion in the medulla oblongata and cerebellum on the left side; the patient was outside the therapeutic window for intravenous thrombolysis and was transferred to our stroke unit. Laboratory results showed normal renal and liver function as well as a normal complete blood count. Total cholesterol was 242 mg/dl and the low-density lipoprotein (LDL) level was 161 mg/dl; the patient was put on statin treatment for secondary prophylaxis. Antihypertensive therapy was started with ramipril.

**Fig. 1:**
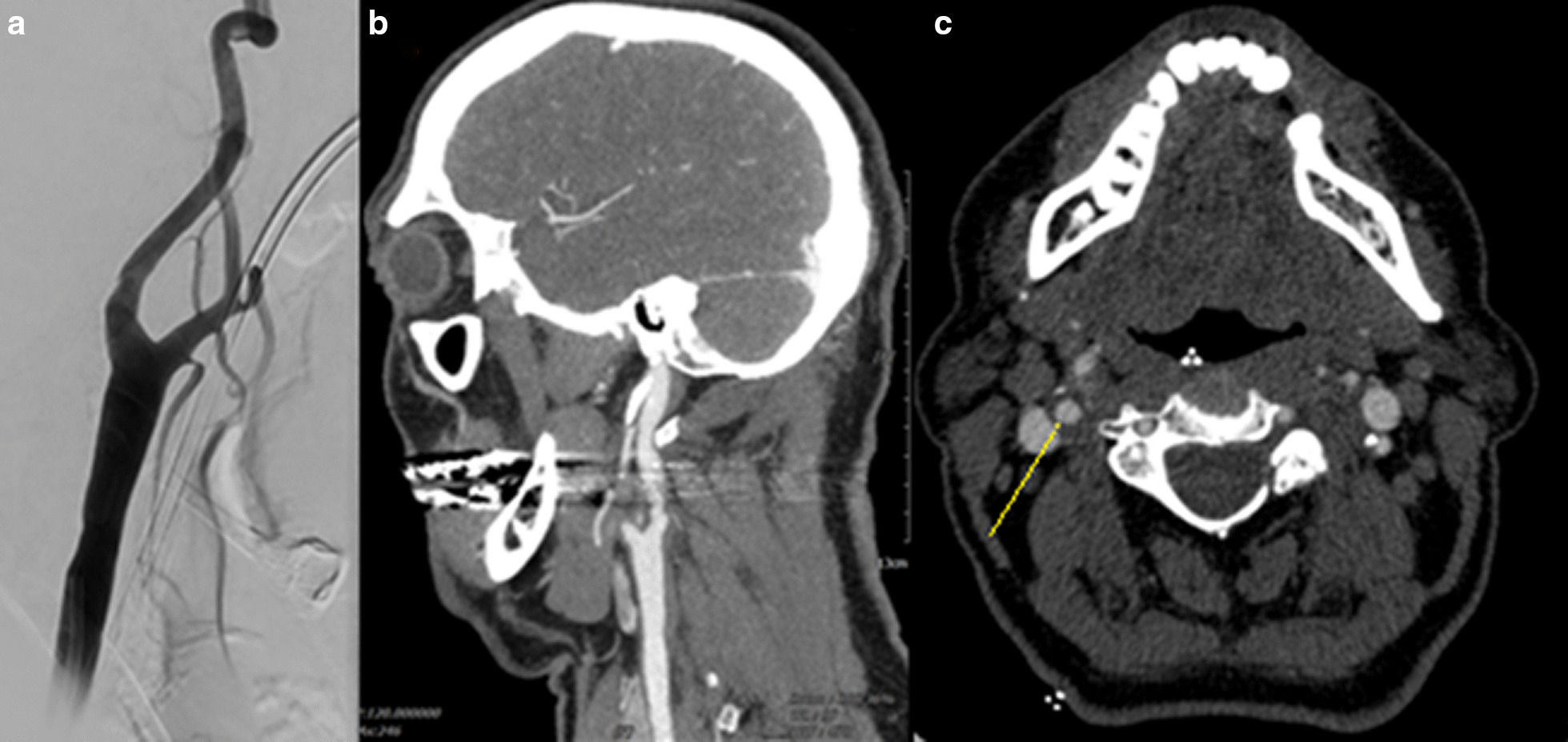
**a** Digital subtraction angiography of the right internal carotid artery. **b** Sagittal view of mild internal carotid artery stenosis. **c** Axial view of the CT-angiogram, the yellow bar shows a carotid web in the right internal carotid artery. With permission of Prof. Ringelstein, Department of Radiology and Neuroradiology, Kliniken Maria Hilf

Approximately 12 hours after admission, the patient reported nonspecific dizziness and blurry vision; the telemetry monitoring showed bradycardia of 30 beats per minute. A new neurological examination showed no new deficits. After a few minutes, and in the presence of the medical team in the patient’s room, the patient started to massage the right side of his neck with intense circular movements; the telemetry again showed bradycardia of 30 beats per minute, and after a few seconds it showed asystole for 4 seconds. The patient stated that he had been suffering from moderate neck pain on the right side for approximately 2 weeks, which he was able to relieve with self-massage. A few minutes later, the patient developed mild left sensorimotor hemisyndrome. A computed tomography (CT) scan showed no new infarct demarcation, and CT-angiography showed plaques in the right internal carotid artery (ICA) consistent with mild right ICA stenosis (Fig. 1b); the flow of the right middle cerebral artery was normal, and the CT axial view showed a membrane in the ICA consistent with a carotid web (Fig. 1c). A carotid dissection was ruled out. Duplex sonography of the carotid showed a flow acceleration up to 187 cm/second, indicating 40% stenosis according to the North American Symptomatic Carotid Endarterectomy Trial (NASCET) criteria. Transesophageal echocardiography showed no pathology. Electrocardiographic monitoring in the stroke unit revealed no atrial fibrillation.

**Fig. 2:**
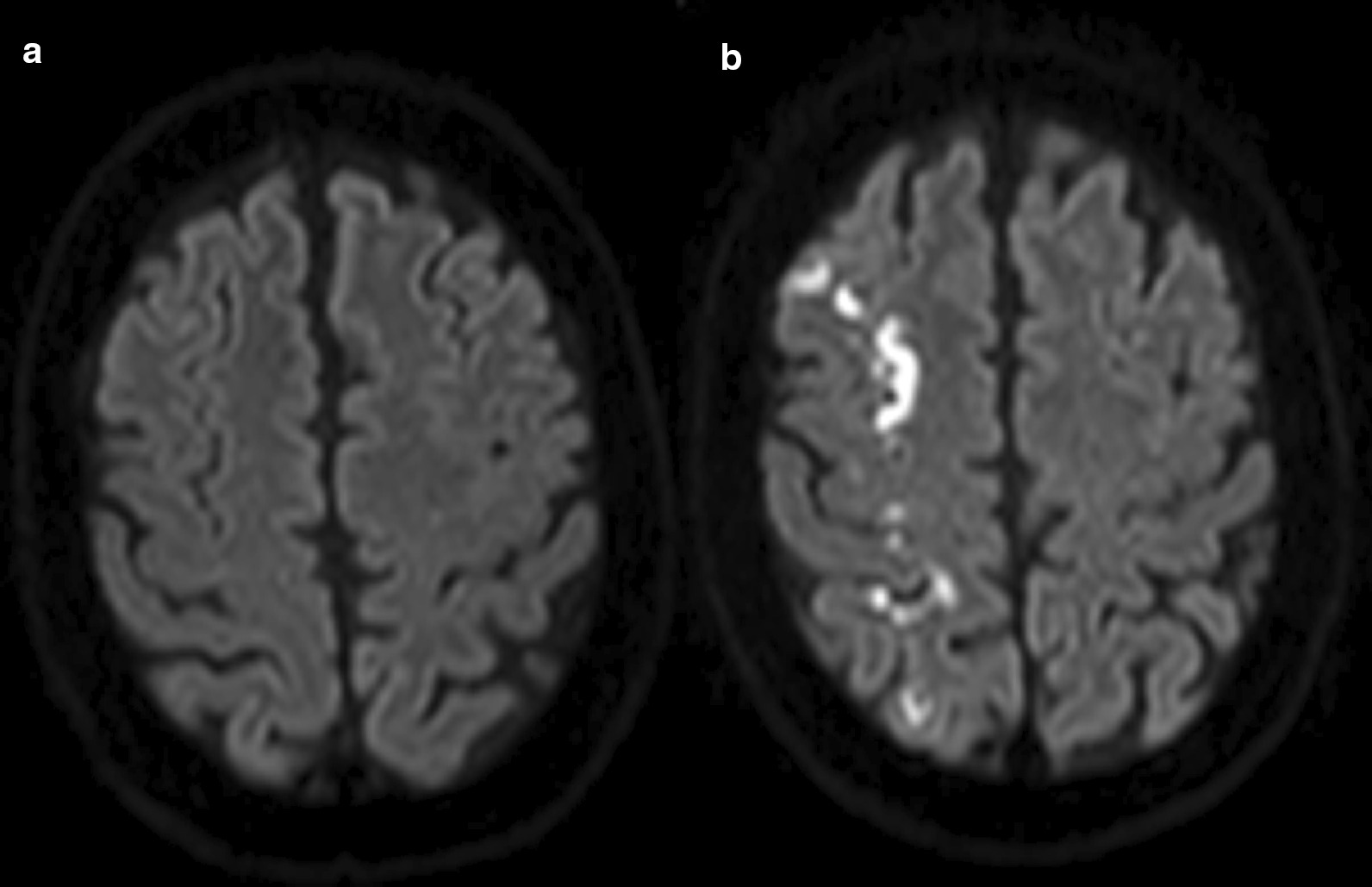
**a** Diffusion-weighted imaging (DWI) on day of admission. **b** DWI after neck massage and asystole. With permission of Prof. Ringelstein, Department of Radiology and Neuroradiology, Kliniken Maria Hilf

The patient underwent MRI the following day, which showed a watershed-type stroke on the right side (Fig. 2).

The patient underwent diagnostic digital subtraction angiography, which showed the known carotid stenosis and carotid web (Fig. 1a). Given the risk of recurrent stroke with a carotid web, the patient received a carotid stent.

The patient was discharged with mild dysphonia due to the primary brainstem infarct.

## Discussion and conclusions

We present the case of a patient who was admitted to our hospital due to a left brainstem and cerebellar infarct of atherosclerotic origin. During his stay in our stroke unit, he performed a self-massage on the right side of his neck, causing stimulation of the CS and leading to a 4-second asystole, resulting in cerebral hypoperfusion. We documented a watershed stroke on an MRI after the massage. During the diagnostic workup, an ultrasound revealed 40% stenosis of the right internal carotid artery, and a CT angiogram revealed mild right ICA stenosis and a carotid web. Based upon the prothrombotic risk of the carotid web, the patient underwent carotid artery stenting and after a few days was discharged. To the best of our knowledge, this is the first documented case of self-massage by a patient with a carotid web and mild stenosis leading to a watershed-type stroke.

The CS and the carotid body are two specialized organs in the wall of the carotid bifurcation; the CS works as a baroreceptor and the carotid body works as a chemoreceptor relaying information about the chemical composition of the arterial blood [2]. They are innervated by the glossopharyngeal and vagus nerve, respectively [3]. The function of the CS is the regulation of the blood pressure. Hypertension stimulates efferent impulses to the vasomotor center in the medulla, inhibiting sympathetic tone and increasing vagal tone [2, 4]. The carotid body works as a baroreceptor monitoring changes in oxygen and carbon dioxide levels [4]. It also induces bradycardia when manipulated (for example during carotid massage) [3].

There are some well-documented cases of secondary ischemic stroke after neck manipulation or massage and arterial dissection (mostly involving the vertebral artery, followed by the carotid artery) [5–9]. The postulated mechanism of massage-associated complications is traumatic fissuring of atherosclerotic plaques in the carotid artery endothelium [10]. Another mechanism of neurological deterioration after CS massage is the dilation of the muscle vascular bed resulting in hypotension.

Tan *et al*. published a case where a male patient developed embolic stroke after self-massage, but the etiology of this stroke was a free-floating thrombus found on the internal carotid artery [1].

**Fig. 3:**
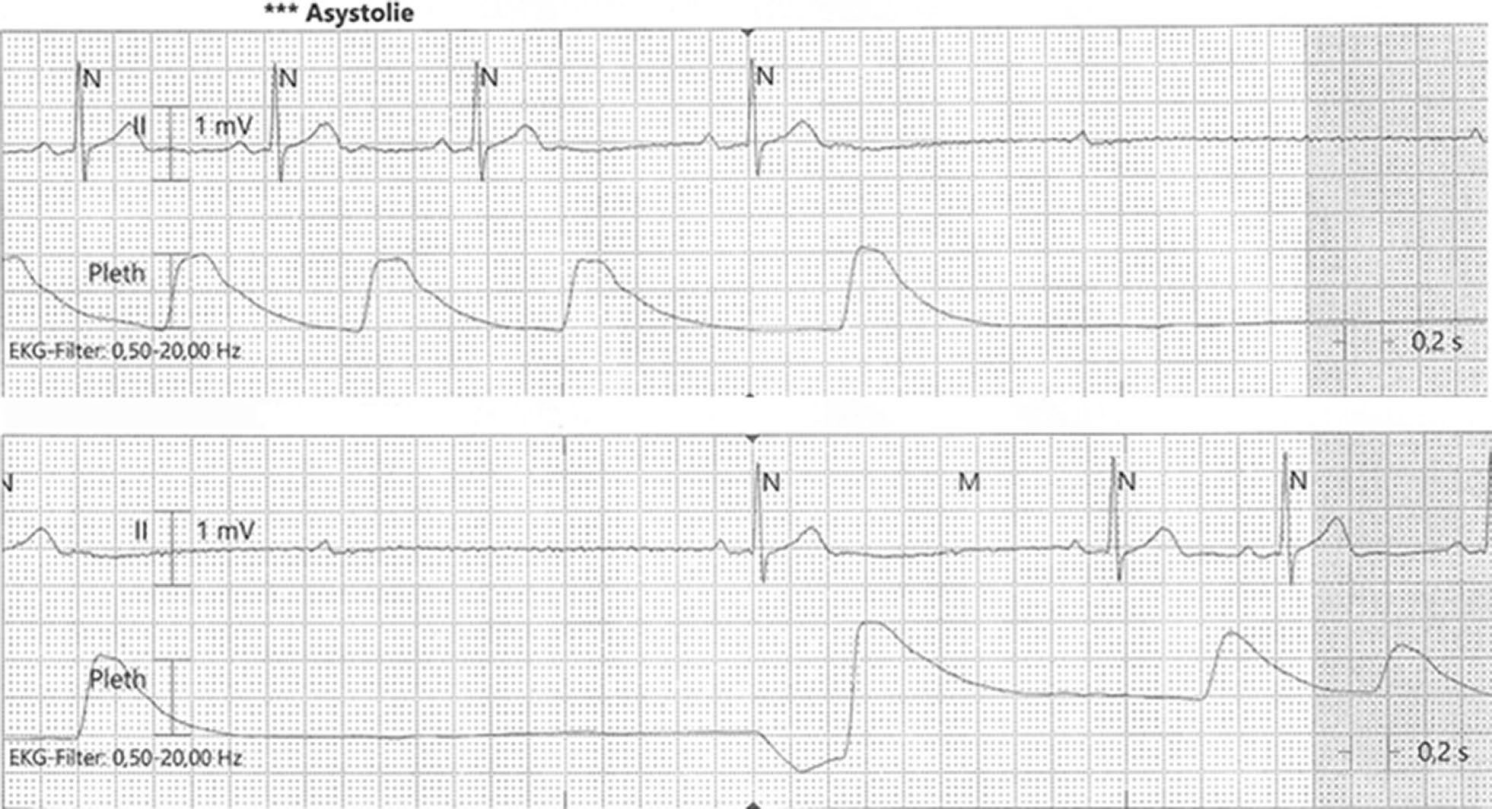
Electrocardiogram showing asystole during the neck massage. With permission of Prof. Haensch, Department of Neurology, Kliniken Maria Hilf

Our patient stated that he had had pain on the right side of his neck that he could successfully treat by neck massage. By the time he was admitted to our stroke unit, the patient had the same neck pain again and massaged his neck to relieve it. The monitor alarm showed bradycardia (30 beats per minutes) and 4-second asystole (Fig. 3). Subsequently, the patient developed dizziness and vegetative symptoms, compatible with presyncope. The patient was immediately advised to stop the massage, and after a few seconds the heart rate normalized. Clinical examination of the neck by palpation showed some bilateral lymph nodes, which were confirmed on the CT scan. An ear-nose-throat (ENT) consultation was performed and did not show any pathologic lymph nodes, possibly due to a past infectious process. Conservative treatment was recommended without further interventions.

We postulate that the repetitive impaired cardiac output, which resulted from the massage, led to the hemodynamic stroke (watershed stroke), which is compatible with the infarction pattern that we saw on subsequent MRI scans.

Take-away lessons:

- Neck massage should not be performed by patients.
- Neck massage/manipulation continues to be a cause of stroke to consider.
- Ischemic stroke after CS and carotid body manipulation is due to thromboembolism by rupture of a plaque or by hypoperfusion due to repetitive hypotension.
- Non-hemodynamically relevant carotid stenosis may be treated in the presence of concomitant symptomatic infarct.

## Data Availability

Not applicable.

## Abbreviations

CS: Carotid sinus
ICA: Internal carotid artery
ENT: Ear-nose-throat (otorhinolaryngology)
